# An Atypical Presentation of an Uncommon Malignancy: Plasmablastic Lymphoma Presenting As Recurrent Scrotal Abscesses

**DOI:** 10.7759/cureus.38879

**Published:** 2023-05-11

**Authors:** Layton Wiemer, JR Quan, Reeba Omman

**Affiliations:** 1 Internal Medicine, University of Florida College of Medicine – Jacksonville, Jacksonville, USA; 2 Oncology, University of Florida College of Medicine – Jacksonville, Jacksonville, USA; 3 Oncology, University of Florida Health, Jacksonville, USA; 4 Pathology, University of Florida College of Medicine – Jacksonville, Jacksonville, USA

**Keywords:** epoch, ebv associated lymphoma, immunocompromised individuals, genitourinary plasmablastic lymphoma, extraoral plasmablastic lymphoma

## Abstract

Plasmablastic lymphoma (PBL) is a rare and extremely diagnostically challenging entity. We report a unique case of PBL in an adult male with a history of recurrent scrotal abscesses who presented with progressively worsening scrotal pain, swelling, and drainage. Pelvic CT demonstrated a large scrotal abscess with external draining tracts with foci of air. Surgical debridement revealed necrotic tissue throughout the abscess cavity, abscess wall, and scrotal skin. Immunohistochemical analysis of the scrotal skin specimen uncovered diffuse proliferation of plasmacytoid cells with immunoblastic features that stained positive for CD138, CD38, IRF4/MUM1, CD45, lambda restriction, and Epstein-Barr encoded RNA in situ hybridization (EBER-ISH) with high Ki-67 proliferation index greater than 90%. Taken together, these findings confirmed a diagnosis of PBL. Treatment with six cycles of infusional etoposide, prednisolone, vincristine, cyclophosphamide, and hydroxydaunorubicin (EPOCH-like regimen) was administered with subsequent positron emission tomography (PET)/CT confirmation of complete response. There was no clinical evidence of lymphoma recurrence at the time of follow-up six months later. Our case exemplifies the growing diversity of ways in which PBL may manifest and underscores the importance of a clinician’s familiarity with this entity and its well-defined risk factor of immunosuppression.

## Introduction

Plasmablastic lymphoma (PBL) is an uncommon type of diffuse large B-cell lymphoma (DLBCL) that disproportionately affects individuals with underlying immunosuppression [1]. Specifically, this disease entity is most well documented in individuals with human immunodeficiency virus (HIV)/acquired immune deficiency syndrome (AIDS) [2]. More recently, PBL has also been identified in other patient populations including individuals on immunosuppressive medications after solid organ transplantation and, albeit infrequently, amongst immunocompetent persons [3,4]. Although the exact incidence of PBL is unknown due to the scarcity of reported cases of this disease, it is estimated that PBL accounts for roughly 2% of all HIV-related lymphoma cases [5].

According to a literature review by Castillo et al. from 1997 to 2014 that analyzed 590 cases of PBL, the three most common sites of involvement were the oral cavity, the gastrointestinal tract, and the lymph nodes [1]. Cumulatively, disease involving these three sites comprised nearly two-thirds of all cases of PBL examined. The two most common sites of disease involvement, the oral cavity and the gastrointestinal tract, together encompassed greater than half of cases of PBL in this study. Consequently, it is unsurprising that weight loss is frequently cited as the most common presenting symptom of this malignancy. Despite PBL having a propensity for arising in specific anatomic locations, increasing numbers of cases have recently been reported with atypical presentations with respect to location and symptomatology. This is highlighted by recently documented cases of PBL involving the lung, peritoneal cavity, left ventricle of the heart, and others [6-8]. We present, to our knowledge, the first reported case of PBL manifesting as recurrent scrotal abscesses secondary to neoplastic infiltration of the scrotal wall.

## Case presentation

A 44-year-old male with a past medical history of nicotine use (active smoker with a 15-pack-year smoking history) and recurrent scrotal abscesses presented to the emergency department with a chief complaint of scrotal swelling, pain, and ulcerations. Over the span of the preceding six years, the patient had presented to the hospital on numerous occasions for groin pain and abscesses on the scrotal wall and proximal thigh requiring incision and drainage. At the time of the current presentation, the patient stated that he had been experiencing chronic scrotal wounds for several years that he would manage at home with topical antibiotics and pain medication, but that over the preceding few weeks the pain and swelling had grown intolerable. Additionally, he mentioned that he noticed new onset of thick yellow-colored drainage from his scrotum, which he had not previously observed. He endorsed accompanying symptoms of subjective fevers and generalized malaise. He specifically denied any weight loss or night sweats.

**Figure 1:**
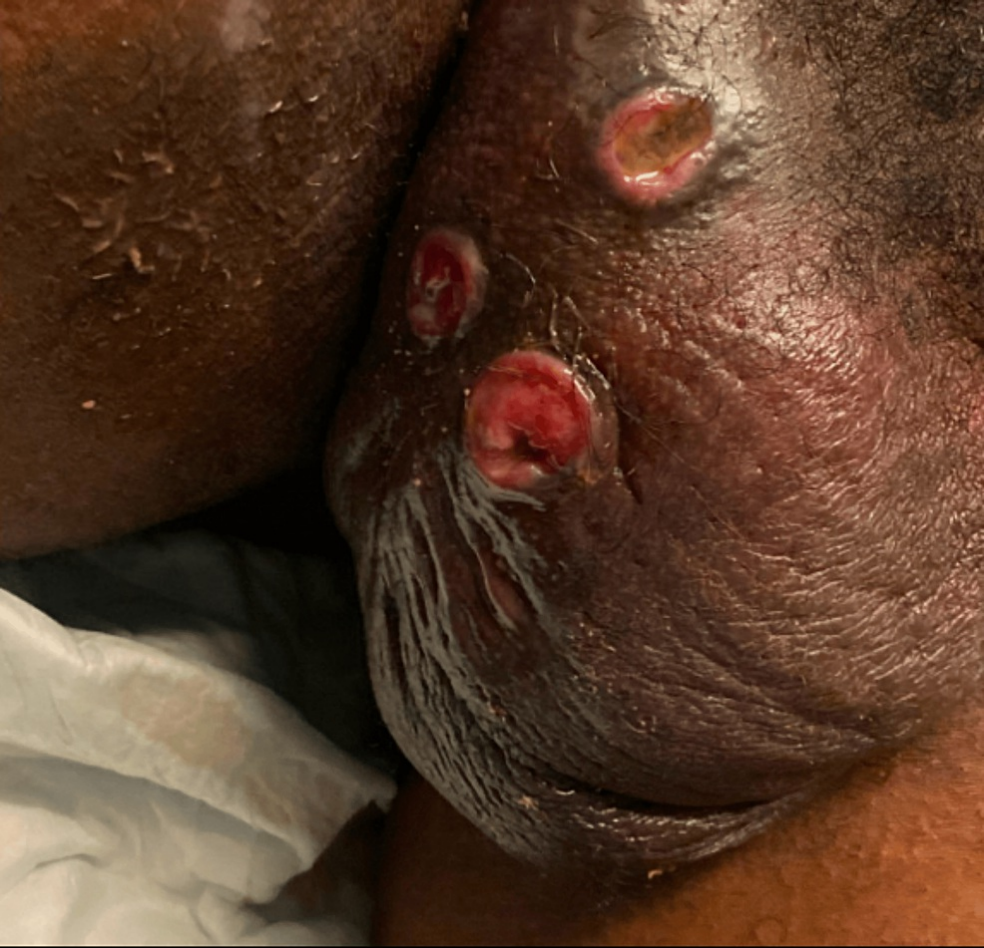
Genitourinary exam findings on arrival to the emergency department.

Physical exam revealed an uncomfortable thin male with an acutely ill appearance. The patient’s vital signs showed that he was afebrile and normotensive, but tachycardic with a heart rate of 120 beats per minute. Cardiorespiratory exam was remarkable for sinus tachycardia without murmurs, rubs, or gallops and lungs were clear to auscultation bilaterally. The patient’s abdomen was soft and flat without tenderness, distention, or masses. Lymph node survey detected palpable painful lymphadenopathy in the bilateral inguinal lymph node chains with the absence of appreciable cervical or supraclavicular lymph node abnormalities. Genitourinary exam revealed a swollen right hemiscrotum with multiple draining wounds and without palpable crepitus, as shown in Figure 1.

Complete blood count and basic metabolic panel were obtained demonstrating no abnormalities. Testing for sexually transmitted diseases was pursued with nucleic acid amplification testing for *Neisseria gonorrhoeae *and *Chlamydia trachomatis* resulting in negative, *Treponema pallidum* antibody testing resulting in non-reactive, and HIV 1/2 antigen/antibody testing resulting in positive for HIV-1. The patient denied a prior diagnosis of HIV or other sexually transmitted diseases. Further evaluation discovered an elevated HIV-1 viral load of 66,400 copies/mL and a reduced CD4 count of 113 cells/mm^3^, which confirmed the patient as being newly diagnosed with HIV.

CT scan of the pelvis demonstrated a large right scrotal abscess measuring 5.0 x 3.6 x 7.0 cm with associated scrotal edema, reactive lymphadenopathy within the groin and pelvis, scattered locules of gas, as well as at least two sinus tracts with drainage to the superficial skin. At this point, urology was consulted and decided to take the patient for debridement and exploration at the site of the genitourinary infection under general anesthesia. Intraoperatively, the patient was found to have three sinus tracts over the right hemiscrotum with very foul-smelling discharge. An incision was made to connect the two medial tracts and open the cavity, which revealed necrotic tissue throughout the abscess cavity, the abscess wall, and within the scrotal skin. The scrotal skin containing the abscess and sinuses and necrotic tissue was incised and the skin and necrotic subcutaneous tissue were removed and sent for histologic analysis.

**Figure 2:**
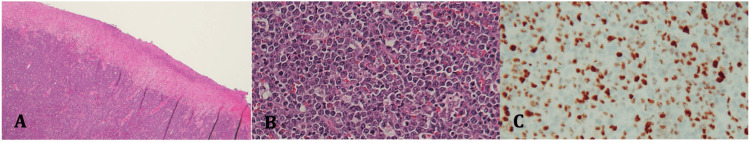
(A) Ulcerated scrotal skin overlying a diffuse proliferation of neoplastic cells. (B) Neoplastic plasmacytoid cells with immunoblastic features. (C) Staining of neoplastic cells to EBER-ISH consistent with diffuse positivity for EBV. EBER-ISH: Epstein-Barr virus-encoded RNA by in situ hybridization; EBV: Epstein-Barr virus

Immunohistochemical (IHC) analysis showed a diffuse and cohesive proliferation of plasmacytoid cells with moderate cytoplasm and cells with immunoblastic features. IHC stains showed these cells were positive for CD138, CD38, CD45, IRF4/MUM1, and lambda restriction. Staining was negative for CD30, CD20, PAX-5, Bcl-6, Bcl-2, ALK-1, and HHV8. Proliferation index (Ki67) was markedly elevated to >90% and the cells stained diffusely positive for Epstein-Barr virus-encoded RNA by in situ hybridization (EBER-ISH). Taken together, these histologic findings highlighted in Figure 2A-2C confirmed a diagnosis of plasmablastic lymphoma.

Staging of the malignancy was subsequently pursued. Bone marrow biopsy with flow cytometry was performed with results showing no overt evidence of immunophenotypic abnormality or neoplasia. MRI of the brain demonstrated no CNS involvement of the lymphoma. CT scans of chest/abdomen/pelvis reported intraabdominal and inguinal lymphadenopathy. Serum protein electrophoresis, serum immunofixation, and kappa/lambda ratio were all within normal limits. He was ultimately diagnosed with Ann Arbor stage IIb PBL. Staging was based on the involvement of multiple lymph node regions, all confined to below the diaphragm, accompanied by B symptom of recurrent unexplained fever > 38^o^C that the patient reported was present both before and after treatment of his scrotal infection.

Amoxicillin-clavulanate and sulfamethoxazole-trimethoprim were co-administered for the treatment of the patient’s acute genitourinary infection based on wound cultures growing methicillin-resistant *Staphylococcus aureus* (MRSA), *Bacteroides fragilis*, and *Peptostreptococcus* species. Simultaneously, he was started on highly active antiretroviral therapy (HAART) for his newly diagnosed HIV infection. After the resolution of the patient’s scrotal infection with antimicrobial therapy, the patient was started on intravenous chemotherapy with etoposide, prednisolone, vincristine, cyclophosphamide, and hydroxydaunorubicin. He underwent a total of six cycles of chemotherapy which were well tolerated except for the development of peripheral neuropathy, which required a dose reduction of vincristine for cycles five and six of his treatment.

Two months following the completion of six cycles of chemotherapy, positron emission tomography (PET)/CT was obtained which confirmed a complete response to treatment with the absence of pathologic fluorodeoxyglucose (FDG) avidity. Given the absence of clinical or imaging evidence of recurrent lymphoma, an active surveillance strategy was exercised. At the most recent follow-up, which was approximately six months following the completion of chemotherapy, the patient remained with no clinical evidence of disease recurrence.

## Discussion

Plasmablastic lymphoma was diagnosed in our patient as an unexpected finding of a scrotal skin debridement that was intended to identify the etiology of genitourinary infection. This case highlights one element of the diagnostic difficulty of this uncommon malignancy in that PBL likely has a predilection for involvement of a wider variety of anatomic locations than previously thought. As reported by Castillo et al. in their 2015 review article, only 23 of the 590 cases of PBL discovered in their literature review had a genitourinary site of involvement and only 41 of the 590 cases of PBL had a cutaneous site of involvement [1]. In our review of the literature via a search for “genitourinary plasmablastic lymphoma” on PubMed, only four cases of genitourinary PBL could be identified. These included two patients with testicular PBL, one patient with PBL of an uncircumcised penile prepuce, and an additional patient with PBL of the uterus [9-12]. Our patient’s case is the first report of PBL manifesting as recurrent scrotal abscesses secondary to neoplastic infiltration of the scrotal wall. This novel presentation underscores the need for increased vigilance on the part of providers by including PBL in the differential diagnosis for individuals with underlying immunosuppression presenting with any cutaneous or genitourinary abnormality. Furthermore, the long history of recurrent infections at the site of development of PBL in our patient warrants further investigation into whether chronic B cell stimulation mediated by bacterial and viral infections could play a role in the incompletely understood pathogenesis of PBL.

Further complicating the diagnosis of this rare entity is the overlap between the clinical and laboratory characteristics of PBL and various other hematologic malignancies including plasmablastic myeloma and other large B cell lymphomas [13]. PBL is typically visualized on histology sections as a diffuse proliferation of large neoplastic cells with a “plasmablast-like” appearance including abundant basophilic cytoplasm and large and irregularly placed nuclei [14]. Despite the similarities in presenting symptoms and tumor cell morphology between PBL and other hematologic malignancies, PBL can be distinguished based on its distinct immunophenotypic footprint. The typical immunophenotype in PBL includes positivity for CD38, CD138, MUM1/IRF4, and EBER accompanied by a high Ki-67 proliferation index [1,5,15]. As demonstrated by our patient’s case, even an atypical presentation of an uncommon pathology can be readily identified by tissue sampling followed by careful analysis of cell morphology and immunophenotype in conjunction with an experienced pathologist.

Given the low incidence of PBL, no prospectively randomized trial has established a definitive first-line treatment regimen for this disease. According to the most recent National Comprehensive Cancer Network Clinical Practice Guidelines in Oncology (NCCN Guidelines®), the preferred treatment option for PBL is etoposide, prednisone, vincristine, cyclophosphamide, and doxorubicin; this regimen is frequently referred to as EPOCH [16]. Alternative regimens such as cyclophosphamide, doxorubicin, vincristine, and prednisolone (CHOP) and cyclophosphamide, vincristine, doxorubicin, and dexamethasone alternating with high-dose methotrexate and cytarabine (hyperCVAD) have been utilized for patients with PBL. However, the NCCN Guidelines® specifically state that treatment with CHOP is not adequate therapy and recommend hyperCVAD as an “other recommended regimen” rather than a “preferred regimen” [16]. These recommendations are supported by findings of a recently published multi-institution retrospective study of outcomes of patients with limited-stage PBL by Hess et al. that found “frontline therapy with EPOCH based regimens showed improved progression-free survival (PFS) outcomes versus CHOP based regimens that were statistically significant in multivariate regression analysis” [17].

Our patient received six cycles of an EPOCH-like regimen based on the aforementioned guidelines and our belief that the inherently aggressive nature of PBL warranted a more dose-intensive approach. After completion of chemotherapy, there was no clinical or radiographic evidence of lymphoma recurrence. Although achieving complete remission of disease with EPOCH is often attainable, unfortunately, these patients relapse early and often, and long-term prognosis remains poor in most cases. An analysis by Makady et al. of 173 patients with PBL, 50% of which were treated with CHOP and 50% of which were treated with more aggressive chemotherapy such as EPOCH, showed a two-year overall survival of 47.4% [18]. The unfavorable prognosis in patients with PBL is most frequently attributed to high rates of relapse and chemotherapy refractoriness. For this reason, active surveillance with follow-up at a minimum every three months was recommended for our patient following the completion of chemotherapy after confirming a complete response.

## Conclusions

Our case adds to the growing pool of evidence that PBL has a more heterogenous presentation with respect to both the site of involvement and the symptomatology than previously thought. Additionally, this case substantiates the importance of identifying underlying immunosuppression as a major risk factor for PBL and consequently pursuing HIV screening whenever a diagnosis of PBL is made. Despite recent advances in the diagnosis and treatment of PBL, long-term outcomes remain poor for this very aggressive disease. Increasing awareness by clinicians of the multitude of ways in which PBL may initially emerge coupled with a better understanding of its risk factors, such as immunosuppression, may allow for earlier diagnosis and more opportunities for initiating guideline-directed treatment for this aggressive lymphoma.

## References

[1] Castillo JJ, Bibas M, Miranda RN (2015). The biology and treatment of plasmablastic lymphoma. Blood.

[2] Castillo JJ, Winer ES, Stachurski D (2010). Clinical and pathological differences between human immunodeficiency virus-positive and human immunodeficiency virus-negative patients with plasmablastic lymphoma. Leuk Lymphoma.

[3] Li YJ, Li JW, Chen KL (2020). HIV-negative plasmablastic lymphoma: report of 8 cases and a comprehensive review of 394 published cases. Blood Res.

[4] Cattaneo C, Facchetti F, Re A (2005). Oral cavity lymphomas in immunocompetent and human immunodeficiency virus infected patients. Leuk Lymphoma.

[5] Carbone A (2002). AIDS-related non-Hodgkin's lymphomas: from pathology and molecular pathogenesis to treatment. Hum Pathol.

[6] Lin Y, Rodrigues GD, Turner JF, Vasef MA (2001). Plasmablastic lymphoma of the lung: report of a unique case and review of the literature. Arch Pathol Lab Med.

[7] Olofson AM, Loo EY, Hill PA, Liu X (2017). Plasmablastic lymphoma mimicking carcinomatosis: a case report and review of the literature. Diagn Cytopathol.

[8] Miller DV, Mookadam F, Mookadam M, Edwards WD, Macon WR (2007). Primary cardiac plasmablastic (diffuse large B-cell) lymphoma mimicking left ventricular aneurysm with mural thrombus. Cardiovasc Pathol.

[9] Sun J, Medeiros LJ, Lin P, Lu G, Bueso-Ramos CE, You MJ (2011). Plasmablastic lymphoma involving the penis: a previously unreported location of a case with aberrant CD3 expression. Pathology.

[10] Nwanwene K, Khan NA, Alsharedi M (2021). Testicular plasmablastic lymphoma in an HIV-negative patient: a rare case presentation. J Investig Med High Impact Case Rep.

[11] Sugimoto K, Koike H, Esa A (2011). Plasmablastic lymphoma of the right testis. Int J Urol.

[12] Matsuki E, Miyakawa Y, Asakawa S (2011). Identification of loss of p16 expression and upregulation of MDR-1 as genetic events resulting from two novel chromosomal translocations found in a plasmablastic lymphoma of the uterus. Clin Cancer Res.

[13] Pileri SA, Mazzara S, Derenzini E (2021). Plasmablastic lymphoma: one or more tumors?. Haematologica.

[14] (2017). WHO Classification of Tumours of Haematopoietic and Lymphoid Tissues. WHO Classification of Tumours of Haematopoietic and Lymphoid Tissues.

[15] Bailly J, Jenkins N, Chetty D, Mohamed Z, Verburgh ER, Opie JJ (2022). Plasmablastic lymphoma: an update. Int J Lab Hematol.

[16] (2023). NCCN Guidelines: B-cell lymphomas. https://www.nccn.org/guidelines/guidelines-detail?category=1&id=1480.

[17] Hess BT, Giri A, Park Y (2023). Outcomes of patients with limited-stage plasmablastic lymphoma: a multi-institutional retrospective study. Am J Hematol.

[18] Makady NF, Ramzy D, Ghaly R, Abdel-Malek RR, Shohdy KS (2021). The emerging treatment options of plasmablastic lymphoma: analysis of 173 individual patient outcomes. Clin Lymphoma Myeloma Leuk.

